# Hypertension and depression coexistence and its association with frailty in older adults: a cross-sectional study

**DOI:** 10.1590/1980-5764-DN-2026-0482

**Published:** 2026-08-24

**Authors:** María Lucia Mansilla-Rivero, Pedro Jorge Lino-Pomasoncco, Lyanne Valery López-Cosme, Alvaro Micael Ñaña-Cordova, José Francisco Parodi, Fernando Miguel Runzer-Colmenares

**Affiliations:** 1 Universidad Científica del Sur, Carrera de Medicina Humana, Lima, Peru.; 2 Universidad Científica del Sur, Carrera de Medicina Humana, CHANGE Research Working Group, Lima, Peru.; 3 Universidad de San Martín de Porres, Facultad de Medicina Humana, Centro de Investigación del Envejecimiento, Lima, Peru.

**Keywords:** Frailty, Hypertension, Depression, Aged, Fragilidad, Hipertensión, Depresión, Anciano

## Abstract

Frailty syndrome is a common condition in older adults, characterized by a decline in physiological reserves and increased vulnerability to stressors. It is particularly prevalent among individuals with comorbidities such as hypertension and depression.

**Objective:**

To assess the association between the coexistence of hypertension and depressive symptoms and frailty syndrome in older adults.

**Methods:**

This observational, cross-sectional study included 1,492 adults aged ≥60 years from the CEMENA Frailty Study. Depressive symptoms was assessed using the 5-item Yesavage Geriatric Depression Scale. Frailty was measured using the Fried phenotype. Poisson regression models were used to estimate prevalence ratios and their 95% confidence intervals (CI), adjusting for potential confounders such as age, gender, education, and multimorbidity.

**Results:**

Of the 1,492 participants, 24.9% (n=372) were classified as frail, and 26.2% (n=391) had both hypertension and depressive symptoms. Their coexistence was significantly associated with frailty (adjusted prevalence ratio=1.90, 95%CI 1.34–2.68, p<0.001). Other factors, including advanced age, multimorbidity, polypharmacy, anxiety symptoms, and neurocognitive impairment, were also associated with frailty.

**Conclusion:**

The coexistence of hypertension and depressive symptoms was significantly associated with frailty in older adults. This finding highlights the need for an integrated approach to managing both cardiovascular and mental health conditions in geriatric care. Early identification and treatment of these conditions may help mitigate frailty and improve overall health and quality of life in aging populations.

## RESUMEN

El síndrome de fragilidad es una condición frecuente en adultos mayores, caracterizada por una disminución de las reservas fisiológicas y una mayor vulnerabilidad frente a factores estresantes. Es particularmente prevalente entre individuos con comorbilidades como hipertensión y depresión.

**Objetivo:**

Evaluar la asociación entre la coexistencia de hipertensión y síntomas depresivos y el síndrome de fragilidad en adultos mayores.

**Métodos:**

Estudio observacional de corte transversal que incluyó a 1.492 adultos ≥60 años del Estudio de Fragilidad CEMENA. Los síntomas depresivos se evaluaron mediante la Escala de Depresión Geriátrica de Yesavage de 5 ítems. La fragilidad se midió utilizando el fenotipo de Fried. Se utilizaron modelos de regresión de Poisson para estimar las razones de prevalencia (RP) y sus intervalos de confianza (IC) del 95%, ajustando por posibles factores de confusión, como edad, sexo, nivel educativo y multimorbilidad.

**Resultados:**

De los 1.492 participantes, el 24,9% (n=372) fueron clasificados como frágiles y el 26.2% (n=391) presentaron coexistencia de hipertensión y síntomas depresivos. La coexistencia de ambas condiciones se asoció significativamente con la fragilidad (RP ajustada=1,90; IC95% 1,34–2,68, p<0.001). Otros factores, como edad avanzada, multimorbilidad, polifarmacia, síntomas de ansiedad y deterioro neurocognitivo, también se asociaron con la fragilidad.

**Conclusión:**

La coexistencia de hipertensión y síntomas depresivos se asoció significativamente con la fragilidad en adultos mayores. Este hallazgo destaca la necesidad de un enfoque integrado para el manejo de las condiciones cardiovasculares y de salud mental en la atención geriátrica. La identificación y el tratamiento tempranos de estas condiciones pueden contribuir a reducir la fragilidad y mejorar la salud general y la calidad de vida de las poblaciones envejecidas.

## INTRODUCTION

Peru, like other Latin American countries, is experiencing a demographic aging process. According to data from the National Institute of Statistics and Informatics (*Instituto Nacional de Estadística e Informática* — INEI), the population aged over 65 years has increased from 5.7% in 1950 to more than 13.9% in 2024^1^. This demographic shift is associated with various physiological transformations, among which frailty syndrome holds a significant place. Frailty is defined as a clinical state characterized by a decline in physiological reserve and increased vulnerability to stressors due to the dysregulation of multiple systems, such as the neurological, cardiovascular, and immune systems^2^. This condition is associated with a higher risk of adverse outcomes, such as falls, functional decline, and mortality^3^. Globally, frailty affects millions of people, with a prevalence ranging from 12 to 24%, depending on the assessment methodology and the population estudied^4^.

Frailty is a multifaceted concept that can be influenced by various sociodemographic, physiological, behavioral, and pathological factors^5^. Seven key factors have been identified in its development: depression, loneliness, limitations in activities of daily living, risk of malnutrition, dietary inflammatory index score, maximum walking speed, and masticatory dysfunction^6^. Among these, depression has been identified as the strongest predisposing factor (odds ratio – OR=4.66, 95% confidence interval — 95%CI 4.07–5.34), in addition to having a global prevalence of 35.1% (95%CI 30.2–40.4%) among older adults^6,7^.

However, in this population, other conditions also play an important role. Chronic diseases such as diabetes, hypertension, and metabolic syndrome are prominent contributors to the development of frailty^8^. Despite the well-known risks associated with these conditions, their prevalence remains high, with hypertension being the most prevalent, affecting more than 75% of adults aged 75 years old or older^9^.

Frailty significantly alters the clinical approach to hypertension in older adults. Several studies have shown that the coexistence of both conditions increases the risk of mortality and reduces survival^10,11^. Furthermore, among hypertensive older adults, factors such as advanced age, female gender, history of hospitalization, and the presence of depressive symptoms have been identified as key determinants of frailty, highlighting the importance of considering both cardiovascular and mental comorbidities in this process^12^.

Mental health and hypertension have been shown to have a bidirectional association, resulting in lower quality of life, reduced treatment adherence, and increased mortality^13^. In recent years, the relationship between hypertension and depression has gained increasing attention due to the high prevalence of their co-occurrence among older adults, estimated at 29%^14^. Moreover, the simultaneous presence of hypertension and depression has been associated with a significant increase in mortality (aPR=1.524; 95%CI 1.180–1.968)^15^. This finding underscores the urgent need to identify factors related to this interaction and its impact on the health of older adults.

Therefore, the objective of this study was to assess whether the coexistence of hypertension and depressive symptoms is associated with an increased risk of frailty among older adults by analyzing the interaction between these factors and their potential associated factors. This analysis will not only allow a better understanding of the complexity of aging in Peru but will also provide crucial evidence to improve prevention and treatment strategies, promoting a more comprehensive approach to the care of frail older adults.

## METHODS

### Design and population

This was an analytical, cross-sectional, secondary study. An analysis was conducted using the database of the CEMENA FRAILTY Study. This study included a sample of 1,896 participants aged 60 years old and older, consisting of retired naval personnel and their relatives, who were evaluated between 2010 and 2015 at the outpatient clinic or day hospital of the Geriatrics Department at the Naval Medical Center^16^. The variables were assessed by geriatricians trained in research. In the original study, the inclusion criteria comprised participants residing in Metropolitan Lima or Callao, receiving care at the outpatient geriatrics clinic, aged 60 years old or older, including retired naval officers and their families, and those receiving outpatient care from the geriatrics service. Exclusion criteria included participants with a diagnosis of cancer, HIV infection, severe functional dependency, hospitalization, home care, or severe stages of dementia.

### Sample

**Figure 1:**
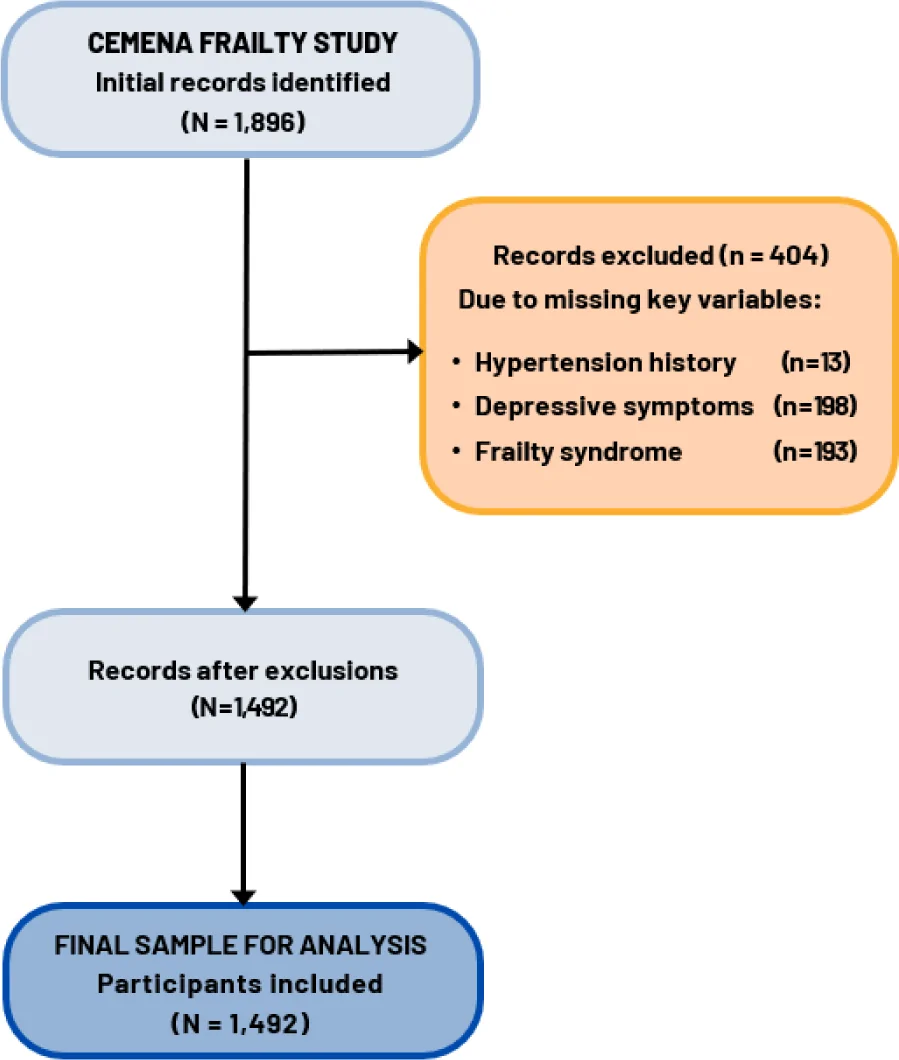
Flowchart of participant selection and exclusions from the CEMENA Frailty Study.

Participant selection in the original study was conducted using a non-probability convenience sampling method. Although convenience sampling was used, the population was large and clinically diverse, which may have mitigated selection bias. Geriatricians recruited participants during outpatient visits and at the outpatient clinic. Information sources included direct interviews, physical examination, indirect medical history, and previous clinical records. For this study, statistical power was calculated using the OpenEpi program, considering an initial sample size of 1,896 participants and a 95%CI (α=0.05), yielding a statistical power of 100%. The information used to estimate statistical power was obtained from a previous study^17^, in which an association between the coexistence of hypertension and depressive symptoms and frailty syndrome was observed. Of the original sample, data from 404 participants were excluded because of missing key variables: history of hypertension (n=13), depressive symptoms (n=198), and frailty syndrome (n=193). After these exclusions, the final sample for analysis consisted of 1,492 participants (Figure 1).

### Main variables

Depressive symptoms were assessed using the Geriatric Depression Scale (GDS-5), with participants scoring two or more considered to have depressive symptoms^18^. Hypertension was defined as a previously established diagnosis obtained from the medical history and diagnosed by the attending physician (cardiologist, internist, or geriatrician). To evaluate the coexistence of hypertension and depressive symptoms, both variables were dichotomized into “yes” and “no.”

The variable “coexistence of hypertension and depressive symptoms” (independent variable) had three categories: none (when the participant had neither hypertension nor depressive symptoms), at least one (when the participant had only one of the two conditions), and both (when the participant had both hypertension and depressive symptoms). The latter category was considered positive only when both variables were present. For statistical analysis, coexistence was defined as the presence of both conditions, whereas the absence of coexistence was defined as having neither condition or only one of the two conditions.

Frailty (dependent variable) was measured using the Fried Frailty Phenotype. A participant was considered frail when meeting three or more of the criteria established by this scale^19^.

### Covariables

Participants’ age was obtained from their medical records and classified into two groups for analysis: ≤79 years and ≥80 years. Gender was considered a dichotomous categorical variable defined as female or male. Educational level was classified into two groups based on years of schooling: participants with fewer than 11 years and those with 11 years or more.

Multimorbidity was defined based on 18 clinical conditions documented in the medical history, including type 2 diabetes mellitus, chronic kidney disease, arterial insufficiency, congestive heart failure, periodontal disease, chronic obstructive pulmonary disease, urinary retention, hypothyroidism, community-acquired pneumonia, stroke, hip fracture, other fractures, osteoarthritis of the hand, knee, or hip, and cervical, lumbar, or hip osteoporosis. For analysis, this variable was dichotomized as “no” (presence of one disease) and “yes” (≥2 diseases).

Polypharmacy was classified according to the number of medications used: participants taking ≤4 medications were classified as “no,” whereas those taking ≥5 medications were classified as “yes”^20^. Anxiety symptoms were assessed using the Hamilton Anxiety Scale; a score of < 14 was considered indicative of the absence of anxiety^20^, and the variable was dichotomized into “does not have” and “has”. Cognitive impairment was evaluated using the Mini-Mental State Examination (MMSE); a score of ≤24 was considered positive and was adjusted for educational level^21^.

### Statistical analysis

Statistical analyses were performed using STATA v18.0 (StataCorp, College Station, TX, USA). Frequencies and percentages of the study variables were calculated to identify significant associations. For the bivariate analysis, the χ^2^ test was used, as recommended for assessing associations between categorical variables when cell frequencies are sufficient, ideally greater than 5. For the multivariate analysis, a Poisson regression model with robust variance was applied. The polypharmacy variable was excluded from the adjusted model due to multicollinearity, evidenced by a variance inflation factor (VIF) greater than 5.

### Ethical considerations

This secondary analysis was approved by the Research Ethics Committee of the Universidad Científica del Sur in Lima, Peru (PRE-15-2024-00027). The primary research, from which the database was derived had previously been approved by the Ethics Committee of CEMENA. All participants provided informed consent during outpatient visits at the Naval Medical Center, ensuring voluntary participation and the protection of confidentiality and anonymity throughout the study.

## RESULTS

From the total of 1,896 participant records initially considered in the CEMENA FRAILTY Study, 404 were excluded from this secondary analysis. Data from 1,492 participants were analyzed. The age group with the highest percentage was 60–79 years, representing 55.29% (n=825). Additionally, most participants were male, accounting for 58.79% (n=877). Furthermore, 76.36% (n=1,127) had ≥11 years of education. A total of 30.82% (n=457) of participants reported taking five or more medications, and 54.16% (n=808) had at least two documented clinical conditions. It is noteworthy that 21.31% (n=318) had chronic obstructive pulmonary disease (COPD), 61.00% (n=916) presented anxiety symptoms, and 41.68% (n=616) had a neurocognitive disorder. A total of 26.21% (n=391) of participants were identified as having coexistence of depressive symptoms and arterial hypertension (Table 1).

**Table 1:** Descriptive and bivariate analysis according to the coexistence of hypertension and depressive symptoms in the study population.

Characteristics	N	%	Coexistence of Depressive symptoms and Hypertension n (%)			p-value*
			No	At least one	Yes	
			n=278 (18.63)	n=823 (55.16)	n=391 (26.21)	
Age (years)						<0.001
≤79	825	55.29	177 (21.45)	428 (51.88)	220 (26.67)	
≥80	667	44.71	101 (15.14)	395 (59.22)	171 (25.64)	
Gender						0.346
Female	615	41.22	118 (19.19)	348 (56.59)	149 (24.23)	
Male	877	58.79	160 (18.24)	475 (54.16)	242 (27.59)	
Education (years)						0.957
<11	365	23.64	63 (18.05)	194 (55.59)	92 (26.36)	
≥11	1,127	76.36	211 (18.72)	624 (55.37)	292 (25.91)	
Polypharmacy (medications)						<0.001
≤4	1,035	69.18	218 (21.25)	634 (61.79)	174 (16.96)	
≥5	457	30.82	56 (12.25)	189 (41.36)	212 (46.39)	
Anxiety Symptoms						<0.001
No	570	38	174 (30.53)	396 (69.47)	0 (00.00)	
Yes	916	61	102 (11.14)	423 (46.18)	391(42.69)	
Cognitive impairment						<0.001
No	862	58.32	196 (22.74)	513 (59.51)	153 (17.75)	
Yes	616	41.68	82 (13.31)	296 (48.05)	238 (38.64)	
Multimorbidity (disease)						0.024
1	684	45.84	138 (21.73)	341 (53.70)	156 (24.57)	
≥2	808	54.16	132 (16.34)	449 (55.57)	227 (28.09)	
Diabetes						<0.001
No	1,240	82.89	205 (16.79)	698 (57.17)	318 (26.04)	
Yes	252	17.11	69 (27.38)	118 (46.83)	65 (25.79)	
Chronic kidney disease						0.006
No	1,213	81.06	204 (17.09)	679 (56.87)	311 (26.05)	
Yes	279	18.94	70 (25.09)	137 (49.10)	72 (25.81)	
Vascular insufficiency						0.013
No	1,429	95.78	272 (19.03)	777 (54.37)	380 (26.59)	
Yes	63	4.22	6 (9.52)	46 (73.02)	11 (17.46)	
Heart failure						0.042
No	1,420	95.17	268 (18.87)	773 (54.44)	379 (26.69)	
Yes	72	4.83	10 (13.89)	50 (69.44)	12 (16.67)	
Periodontal disease						<0.001
No	1,287	86.26	273 (21.21)	636 (49.42)	378 (29.37)	
Yes	205	13.74	5 (2.44)	187 (91.22)	13 (6.34)	
Chronic obstructive pulmonary disease (COPD)						0.301
No	1,174	78.69	218 (18.74)	629 (54.08)	316 (27.17)	
Yes	318	21.31	56 (17.61)	187 (58.81)	75 (23.58)	
Urinary incontinence						0.556
No	1,318	88.34	250 (18.97)	721 (54.70)	347 (26.33)	
Yes	174	11.66	28 (16.09)	102 (58.62)	44 (25.29)	
Hip fracture						0.607
No	1,437	96.31	270 (18.79)	793 (55.18)	374 (26.03)	
Yes	55	3.69	8 (14.55)	30 (54.55)	17 (30.91)	
Other fractures						0.101
No	1,446	96.92	275 (19.02)	794 (54.91)	377 (26.07)	
Yes	46	3.08	3 (6.52)	29 (63.04)	14 (30.43)	
Hypothyroidism						<0.001
No	1,320	88.47	269 (20.38)	743 (56.29)	308 (23.33)	
Yes	172	11.53	9 (5.23)	80 (46.51)	83 (48.26)	
Community-acquired pneumonia						0.039
No	1,253	83.98	247 (19.71)	686 (54.75)	320 (25.54)	
Yes	239	16.02	31 (12.97)	137 (57.32)	71 (29.71)	
Cerebrovascular disease						<0.001
No	1,437	96.27	278 (19.61)	772 (54.44)	368 (25.95)	
Yes	55	3.73	0 (00.00)	32 (58.18)	23 (41.82)	
Hand osteoarthritis						0.001
No	1,280	85.79	249 (19.45)	716 (55.94)	315 (24.61)	
Yes	212	14.21	29 (13.68)	107 (50.47)	76 (35.85)	
Cervical osteoarthritis						0.019
No	1,354	90.75	263 (19.42)	746 (55.10)	345 (25.48)	
Yes	138	9.25	15 (10.87)	77 (55.80)	46 (33.33)	
Lumbar osteoarthritis						0.429
No	1,160	77.75	220 (18.97)	645 (55.60)	295 (25.43)	
Yes	332	22.25	58 (17.47)	178 (53.61)	96 (28.92)	
Hip osteoarthritis						0.058
No	1,315	88.14	256 (19.47)	722 (54.90)	337 (25.63)	
Yes	177	11.86	22 (12.43)	101 (57.06)	54 (30.51)	
Knee osteoarthritis						0.013
No	1,203	80.63	240 (19.95)	661 (54.95)	302 (25.10)	
Yes	289	19.37	38 (13.15)	162 (56.06)	89 (30.80)	
Wrist osteoarthritis						0.001
No	1,408	94.37	249 (19.45)	716 (55.94)	315 (24.61)	
Yes	84	5.63	29 (13.68)	107 (50.47)	76 (35.85)	

Note: *p-values were calculated using the χ^2^ Test.

Table 1 presents the bivariate analysis according to the coexistence of arterial hypertension and depressive symptoms as the main variable. Regarding age, 25.64% (n=171) of participants with coexistence of arterial hypertension and depressive symptoms were aged 80 years old or older. In relation to polypharmacy, 46.39% (n=212) of those with both conditions used more than five medications. Likewise, 42.69% (n=391) presented anxiety symptoms in the context of the coexistence of arterial hypertension and depressive symptoms. With respect to multimorbidity, 28.09% (n=227) of participants had at least two additional diseases besides arterial hypertension and depressive symptoms. Moreover, 38.64% (n=238) of individuals with cognitive impairment also presented arterial hypertension and depressive symptoms. In relation to other comorbidities, 25.79% (n=65) of patients with diabetes mellitus and 25.81% (n=72) of those with chronic kidney disease (CKD) exhibited both conditions. Similarly, vascular insufficiency was associated with arterial hypertension and depressive symptoms in 17.46% (n=11) of cases, as was heart failure in 16.67% (n=12) of participants. In the case of periodontal disease, 6.34% (n=13) of affected participants also presented this combination of conditions. Likewise, hypothyroidism was found in 48.26% (n=83) of individuals with hypertension and depressive symptoms. Additionally, 29.71% (n=71) of patients with community-acquired pneumonia had both conditions concurrently. Similarly, 35.85% (n=76) of individuals with hand osteoarthritis and 33.33% (n=46) of those with cervical osteoarthritis presented the same association. Furthermore, 41.82% (n=23) of patients with cerebrovascular disease (CVD) exhibited the coexistence of arterial hypertension and depressive symptoms, as did 30.80% (n=89) of participants with knee osteoarthritis and 35.85% (n=76) of those with wrist osteoarthritis.

Table 2 presents the bivariate analysis according to frailty syndrome. A total of 24.93% (n=372) of participants were identified as having frailty syndrome, with statistically significant differences observed across variables. Frailty syndrome was present in 32.23% (n=215) of participants aged 80 years old or older, demonstrating greater vulnerability in this age group. Regarding gender, 29.08% (n=255) of male participants also presented this condition. With respect to educational level, 24.07% (n=260) of those with at least 11 years of education exhibited frailty syndrome. In terms of comorbidities, 32.80% (n=265) of participants with two or more diseases were also identified as having this condition. Likewise, 52.74% (n=216) of those taking five or more medications, indicative of polypharmacy, presented frailty syndrome. Vascular insufficiency was associated with frailty in 30.16% (n=19) of cases. In addition, 34.72% (n=318) of participants with anxiety symptoms and 41.07% (n=273) of those with cognitive impairment also presented this condition.

**Table 2:** Bivariate analysis according to frailty syndrome in the study population.

Characteristics	Frailty n (%)		p-value*
	No	Yes	
	n=1,120 (75.07)	n=372 (24.93)	
Age (years)			<0.001
≤79	668 (80.97)	157 (19.03)	
≥80	452 (67.77)	215 (32.23)	
Gender			<0.001
Female	498 (80.98)	117 (19.02)	
Male	622 (70.92)	255 (29.08)	
Education (years)			0.006
<11	243 (69.63)	106 (30.37)	
≥11	867 (76.93)	260 (23.07)	
Multimorbidity (disease)			<0.001
1	536 (84.41)	99 (15.59)	
≥2	543 (67.20)	265 (32.80)	
Polypharmacy (medications)			<0.001
≤4	895 (87.23)	131 (12.77)	
≥ 5	216 (47.26)	241 (52.74)	
Anxiety symptoms			<0.001
No	516 (90.53)	54 (9.47)	
Yes	598 (65.28)	318 (34.72)	
Cognitive impairment			<0.001
No	757 (87.82)	105 (12.18)	
Yes	363 (58.93)	253 (41.07)	
Diabetes			0.13
No	909 (74.45)	312 (25.55)	
Yes	199 (78.97)	53 (21.03)	
Chronic kidney disease			0.894
No	899 (75.29)	295 (24.71)	
Yes	209 (74.91)	70 (25.09)	
Vascular insufficiency			0.327
No	1,076 (75.30)	353 (24.70)	
Yes	44 (69.84)	19 (30.16)	
Heart failure			0.025
No	1,074 (75.63)	346 (24.37)	
Yes	46 (63.89)	26 (36.11)	
Periodontal disease			<0.001
No	936 (72.73)	351 (27.27)	
Yes	184 (89.76)	21 (10.24)	
Chronic obstructive pulmonary disease (COPD)			0.391
No	865 (74.38)	298 (25.62)	
Yes	244 (76.73)	74 (23.27)	
Urinary incontinence			<0.001
No	1,025 (77.77)	293 (22.23)	
Yes	95 (54.60)	79 (45.40)	
Hip fracture			<0.001
No	1,108 (77.11)	329 (22.89)	
Yes	12 (21.82)	43 (78.18)	
Other fractures			0.003
No	1,094 (75.66)	352 (24.34)	
Yes	26 (56.52)	20 (43.48)	
Hypothyroidism			<0.001
No	1,028 (77.88)	292 (22.12)	
Yes	92 (53.49)	80 (46.51)	
Community-acquired pneumonia			<0.001
No	976 (77.89)	277 (22.11)	
Yes	144 (60.25)	95 (39.75)	
Cerebrovascular disease			<0.001
No	1,087 (76.66)	331 (23.34)	
Yes	15 (27.27)	40 (72.73)	
Hand osteoarthritis			<0.001
No	1,009 (78.83)	271 (21.17)	
Yes	111 (52.36)	101 (47.64)	
Cervical osteoarthritis			<0.001
No	1,051 (77.62)	303 (22.38)	
Yes	69 (50.00)	69 (50.00)	
Lumbar osteoarthritis			<0.001
No	915 (78.88)	245 (21.12)	
Yes	205 (61.75)	127 (38.25)	
Hip osteoarthritis			<0.001
No	1,030 (78.33)	285 (21.67)	
Yes	90 (50.85)	87 (49.15)	
Knee osteoarthritis			<0.001
No	954 (79.30)	249 (20.70)	
Yes	166 (57.44)	123 (42.56)	
Wrist osteoarthritis			<0.001
No	1,009 (78.83)	271 (21.17)	
Yes	111 (52.36)	101 (47.64)	

Notes: *p-values were calculated using the χ^2^ Test.

Table 3 presents the regression analysis. The crude analysis showed a strong association between the coexistence of hypertension and depressive symptoms and frailty (crude prevalence ratio — cPR=3.60, 95%CI 2.51–5.16, p<0.001), which decreased after adjustment (adjusted prevalence ratio — aPR=1.90, 95%CI 1.34–2.68, p<0.001). The model was adjusted for age, gender, educational level, and multimorbidity. Anxiety symptoms showed an elevated risk in the crude model (cPR=3.66, 95%CI 2.80–4.79, p<0.001), which was reduced in the adjusted model (aPR=2.23, 95%CI 1.67–2.99, p<0.001). Similarly, cognitive impairment showed a strong association in both the crude model (cPR=3.37, 95%CI 2.75–4.12, p<0.001) and the adjusted model (aPR=2.39, 95%CI 1.97–2.91, p<0.001). Polypharmacy was associated with frailty in the crude model (cPR=4.13, 95%CI 3.44–4.95, p<0.001). However, it was not included in the adjusted model due to a VIF greater than 5, suggesting the presence of multicollinearity. Multimorbidity and age showed associations in both models (aPR=1.45 and 1.41, respectively, p<0.001). In contrast, gender maintained its association (aPR=1.49, p<0.001) after adjustment, whereas education shifted from a protective association in the crude model (cPR=0.75, p=0.005) to a non-significant association after adjustment (aPR=1.07, p<0.001).

**Table 3:** Regression analysis evaluating the association between frailty syndrome and the coexistence of hypertension and depressive symptoms in the study population.

Frailty	Models					
	Crude			Adjusted		
	cPR	95%CI	p-value	aPR	95%CI	p-value
Coexistence of depressive symptoms and hypertension	3.6	2.51–5.16	<0.001	1.9	1.34–2.68	<0.001
Anxiety symptoms	3.66	2.80–4.79	<0.001	2.23	1.67–2.99	<0.001
Cognitive impairment	3.37	2.75–4.12	<0.001	2.39	1.97–2.91	<0.001
Multimorbidity	2.1	1.71–2.58	<0.001	1.45	1.19–1.77	<0.001
Polypharmacy	4.13	3.44–4.95	<0.001	not considered	not considered	not considered
Age	1.69	1.41–2.02	<0.001	1.41	1.20–1.65	<0.001
Gender	1.52	1.25–1.85	<0.001	1.49	1.25–1.78	<0.001
Education	0.75	0.62–0.91	0.005	1.07	0.89–1.29	0.42

Abbreviations: cPR, crude prevalence ratio; aPR, adjusted prevalence ratio; 95%CI, 95% confidence interval.

Notes: *The adjusted model includes the variables: age, gender, education, multimorbidity; *The polypharmacy variable was not considered in the adjusted model due to the presence of multicollinearity.

## DISCUSSION

The objective of this study was to assess whether the coexistence of hypertension and depressive symptoms is associated with an increased likelihood of frailty. The results showed that participants with both conditions had a higher likelihood of being frail compared to those without these conditions. This association remained after adjusting for variables such as age, gender, education level, and multimorbidity. As secondary findings, associations were identified between frailty and mental health conditions, such as anxiety symptoms and cognitive impairment. International evidence supports these findings, which are consistent with the multifactorial nature of frailty syndrome, whereby mental health problems contribute to functional decline and increased vulnerability among older adults^22,23^.

Furthermore, an association between multimorbidity and a higher likelihood of frailty was observed. This finding is consistent with recent analyses reporting a prevalence of 72%, identifying multimorbidity as a significant predictor of mortality^24,25^. In this context, it has been reported that, in addition to multimorbidity, deficits associated with frailty increase the risk of institutionalization and the need for home care^26^. Regarding sociodemographic factors, both age and gender showed significant associations with frailty. In particular, aging is consistently associated with a higher prevalence of this syndrome, a finding corroborated by cohort studies documenting a sustained increase in frailty with advancing age^26,27^. Regarding gender, in contrast to the present findings, previous studies have reported a higher prevalence among women; however, this prevalence varies according to individual characteristics^28^. This disparity may be explained by differences in clinical settings, levels of healthcare, burden of specific comorbidities, and the type of population evaluated, which in this case was related to the military environment, where males have historically predominated. Although educational level showed a protective association in the crude analysis, this effect disappeared after adjustment, suggesting that clinical factors such as multimorbidity may play a more relevant role than educational background in explaining frailty within this military-affiliated population.

The present study identified that the coexistence of hypertension and depressive symptoms was associated with nearly twice the prevalence of frailty, reinforcing the notion that both conditions may act synergistically to increase vulnerability among older adults. This interaction becomes particularly relevant considering that hypertension and depression commonly coexist in older age and that their concurrence has previously been associated with worse outcomes, including lower quality of life, poorer treatment adherence, and a higher risk of mortality^13,14,15^. Additionally, among individuals aged 80 years old or older, the simultaneous presence of both conditions has been associated with a prevalence of frailty up to five times greater^17^. Complementary studies have shown that the burden of hypertension tends to increase as depressive symptoms become more severe^29^. This finding suggests a shared pathophysiological axis that may accelerate the transition to frailty. Taken together, these results underscore the importance of integrating cardiovascular and mental health assessments in geriatric care, as their coexistence appears to play a decisive role in functional decline and progression toward frailty.

A potential underlying mechanism for these findings involves a chronic proinflammatory state shared by hypertension and depression, characterized by elevated levels of interleukin-6 (IL-6) and tumor necrosis factor-alpha (TNF-α), which induce muscle catabolism and endothelial dysfunction, thereby favoring the frailty phenotype^30^. Similarly, in skeletal muscle, increased production of reactive oxygen species reduces the regenerative capacity of satellite cells, promoting sarcopenia and frailty; this mechanism is consistent with the mitochondrial theory of aging^31^. Likewise, the hypothalamic-pituitary-adrenal axis coordinates the stress response, and prolonged depression is associated with chronic hypercortisolism, which induces a catabolic state that favors proteolysis and muscle atrophy^32^. Consequently, sarcopenia and reduced muscle strength develop, both of which are central components of physical frailty and contribute to an increased risk of functional decline^32^. From a clinical perspective, these mechanisms highlight the importance of early screening for depressive symptoms and frailty among hypertensive older adults in primary care settings. Simultaneous identification of these conditions may facilitate timely interventions targeting both cardiovascular and mental health domains, potentially reducing the progression toward frailty.

In addition to biological mechanisms, behavioral and psychosocial pathways may also help explain the observed association. Individuals with depressive symptoms often experience reduced motivation, low self-efficacy, and social withdrawal, which may negatively affect engagement in health-promoting behaviors^33^. These factors may contribute to poorer adherence to antihypertensive treatment, unhealthy dietary patterns, and lower levels of physical activity^33^. Such behaviors may worsen blood pressure control and contribute to progressive functional decline^34^. Collectively, these pathways may further increase vulnerability to frailty, highlighting the multifactorial nature of this condition in older adults.

The findings of this study provide relevant evidence regarding the interaction between hypertension and depressive symptoms as factors that enhance biological vulnerability in older adults, reinforcing the need for an integrated clinical approach that simultaneously considers cardiovascular and mental health. In the context of accelerated population aging in Peru, these results emphasize the urgency of implementing systematic screening strategies for depression and frailty among hypertensive patients, as well as geriatric care models that incorporate multidimensional interventions aimed at reducing the progression to frailty. Future longitudinal studies and intervention trials are needed to clarify the temporal directionality of this association, identify specific biological mechanisms, and assess whether timely treatment of these conditions can modify frailty risk and improve functional outcomes in this population.

This study has several strengths, including the use of a large, clinically diverse sample evaluated with validated instruments by specialized personnel, which enhances data quality and the reliability of the findings. Additionally, the use of adjusted models provided robust estimates and adequate control of potential confounding variables. However, the cross-sectional design precludes the establishment of causal relationships and allows only associative inferences. Clinical information was obtained from medical records, which may have resulted in underreporting of some conditions. Additionally, polypharmacy was excluded from the multivariable model due to multicollinearity (VIF>5), and no formal interaction analysis between hypertension and depressive symptoms was performed beyond the categorical coexistence approach used. Furthermore, the convenience sampling of a military-affiliated population limits the generalizability of the findings to other groups pf older adult. The study population was derived from a military-affiliated healthcare system and may therefore have specific socioeconomic, health-related, and lifestyle characteristics compared with the general older adult population. Factors such as differential access to healthcare, higher levels of discipline or physical activity, and distinct life stressors may have influenced the observed associations and could limit the external validity of the findings. The main independent variable was dichotomized as the coexistence of hypertension and depressive symptoms versus absence of coexistence, precluding evaluation of the independent or additive effects of each condition on frailty. Another limitation is the use of an MMSE cutoff score of ≤24 to identify cognitive impairment. Given that most participants had a relatively high educational level, this threshold may have underestimated cognitive impairment and introduced potential misclassification bias, as higher cutoffs values have been suggested in validation studies conducted in the Peruvian population. Lastly, social desirability bias and recall bias may have been present in the self-reported variables, potentially affecting the accuracy of some measurement.

In conclusion, the coexistence of hypertension and depressive symptoms was associated with a higher likelihood of frailty among older adults, even after adjustment for relevant sociodemographic and clinical factors. This finding highlights the multidimensional nature of frailty and the importance of considering the interaction between chronic diseases and mental disorders in comprehensive geriatric assessment. Early identification of these coexisting conditions may contribute to the development of more effective prevention strategies aimed at preserving autonomy and improving quality of life in this population.

## Data Availability

The datasets generated and/or analyzed during the current study are publicly available at Figshare (https://doi.org/10.6084/m9.figshare.13059011).
